# Temporomandibular disorders and neck disability in individuals with cervical disc herniation

**DOI:** 10.1111/eos.70047

**Published:** 2025-10-28

**Authors:** Turgay Altunalan, Esin Geçgil Nazli, İlayda Gür, Nurhayat Korkmaz Üçüncü

**Affiliations:** ^1^ Faculty of Health Science, Department of Physiotherapy and Rehabilitation Karadeniz Technical University Trabzon Turkey; ^2^ Population Health Sciences Institute Newcastle University Newcastle upon Tyne UK; ^3^ Graduate School of Health Sciences Uskudar University, Physiotherapy and Rehabilitation Istanbul Turkey

**Keywords:** cervical spine, disability, pain, physiotherapy, rehabilitation, temporomandibular joint

## Abstract

The primary aim of this study was to investigate the prevalence and severity of temporomandibular disorders (TMDs), and temporomandibular joint (TMJ) pain in patients with cervical disc herniation. The secondary aim was to investigate the relationship between neck disability, neck pain, sociodemographic factors, TMD, and TMJ pain. The participants were divided based on their Neck Disability Index (NDI) score into those with no/mild (*n* = 26), or moderate/severe (*n* = 31) disability. The severity of TMD was assessed using the Fonseca Anamnestic Index, and TMJ pain was assessed using the Visual Analog Scale. Among participants with no/mild neck disability, 88.5% had TMD; in the moderate/severe group, all had TMD. Participants with moderate/severe neck disability demonstrated a higher level of TMD severity and TMJ pain than participants with no/mild disability. The NDI score and female gender were both associated with higher Fonseca Anamnestic Index scores, and these two factors explained 12.% of the variance. Furthermore, the NDI score, neck pain, and female gender were positively associated with higher TMJ pain, explaining 22.1.% of the variance. A high NDI score and female gender were associated with higher severity of TMD. Our study suggests that individuals with cervical disc herniation who present a high NDI score should also be assessed for TMD.

## INTRODUCTION

Cervical disc herniation is a highly prevalent spinal pathology and a significant source of neck pain in the adult population, with a prevalence of approximately 60%, higher among women and older adults [1]. Cervical disc herniation typically presents with a spectrum of symptoms such as neck pain, radicular sensory disturbances, and muscle weakness in affected regions. An emerging body of evidence highlights a potential association between cervical spine conditions and temporomandibular disorders (TMDs), primarily due to their anatomical proximity and functional interdependence [2]. The reported prevalence of TMD among individuals diagnosed with cervical disc herniation is notably high (approximately 46.9%), whereas cervical disc herniation is found in around 10.7% of individuals with TMD [3, 4].

TMDs refer to musculoskeletal conditions affecting the temporomandibular joint (TMJ) and surrounding structures, often characterized by pain, joint sounds, and restricted mouth opening, impairing essential functions such as chewing and speaking [5]. TMD is highly prevalent, affecting approximately 31% of the adult and elderly population, yet remains poorly recognized in clinical settings; about half of those affected do not seek treatment [5, 6]. Frequently, patients present with neck pain and headaches rather than jaw complaints, which can obscure diagnosis [7]. If left untreated, TMD may become chronic, leading to functional limitations, psychological distress, and diminished quality of life [4, 8]. As the second most common musculoskeletal disorder after low back pain, TMD also poses a growing public health burden. In the United States, annual management costs have surpassed $4 billion, excluding imaging expenses [9]. Early identification and appropriate management of TMD symptoms may enhance patients' overall functional capabilities and psychosocial well‐being, highlighting the clinical importance of timely assessment and intervention.

Anatomical and neurophysiological mechanisms help explain the observed interdependence between cervical disorders and TMD. The close spatial relationship and shared innervation between masticatory and cervical musculature suggest that dysfunction in one system may propagate compensatory patterns or pain in the other. The trigeminocervical nucleus, serving as an anatomical and functional crossroad for nociceptive signals from both the upper cervical spine and trigeminal pathways, may further explain the overlapping symptomatology of TMD and cervical disc herniation [3, 10, 11, 12]. Although current clinical guidelines highlight the importance of cervical spine evaluation in TMD management [13, 14, 15, 16, 17], the reverse—assessing TMD in individuals with cervical pathologies such as cervical disc herniation—has received limited attention [4, 8, 18]. Recognizing this bidirectional relationship is essential; early identification and management of TMD in cervical disc herniation patients could enhance clinical outcomes and prevent chronicity.

Thus, this study aims primarily to evaluate the severity of TMD symptoms and intensity of TMJ‐related pain in patients with cervical disc herniation. Additionally, it seeks to explore associations between TMD and TMJ pain intensity, cervical disability severity, neck pain levels, and relevant sociodemographic factors. We hypothesize that greater cervical disability will be associated with increased severity of TMD and elevated TMJ pain intensity.

## MATERIAL AND METHODS

This study employed a cross‐sectional, observational design. Ethical approval was granted by the Uskudar University Ethics Committee (Approval No: 6135131342, April 28, 2022), and the study adhered to the Declaration of Helsinki guidelines. All participants provided written informed consent. Reporting adhered to the Strengthening the Reporting of Observational Studies in Epidemiology (STROBE) guidelines for cross‐sectional studies [19]. The study was registered at ClinicalTrials.gov (NCT06141863, 11/20/2023).

### Participants

The participants were adults aged 18–65 who had been diagnosed with cervical disc herniation (bulging, protrusion, or extrusion) and had applied to the Fizyotown and Meditan Medical Centers, located in major cities in Turkey. Participants were invited to take part in the study based on their records at the physical therapy clinic between May 2023 to February 2024. Diagnoses of participants were confirmed clinically and through neck magnetic resonance imaging evaluations by a physical medicine specialist. Only participants who had not received prior medical or physiotherapy treatment for their cervical and TMD complaints were included. Other exclusion criteria were as follows: Presence of neurological deficits (loss of strength or sensation in upper extremities), history of cervical fracture or previous head‐neck surgery, diagnosed inflammatory rheumatic diseases (e.g., ankylosing spondylitis, rheumatoid arthritis), history of infection or facial paralysis, advanced osteoporosis, prior diagnosis, or treatment for TMD (including myalgia or arthralgia). Demographic characteristics were recorded and these included age, gender, marital status, education, and income level.

**FIGURE 1:**
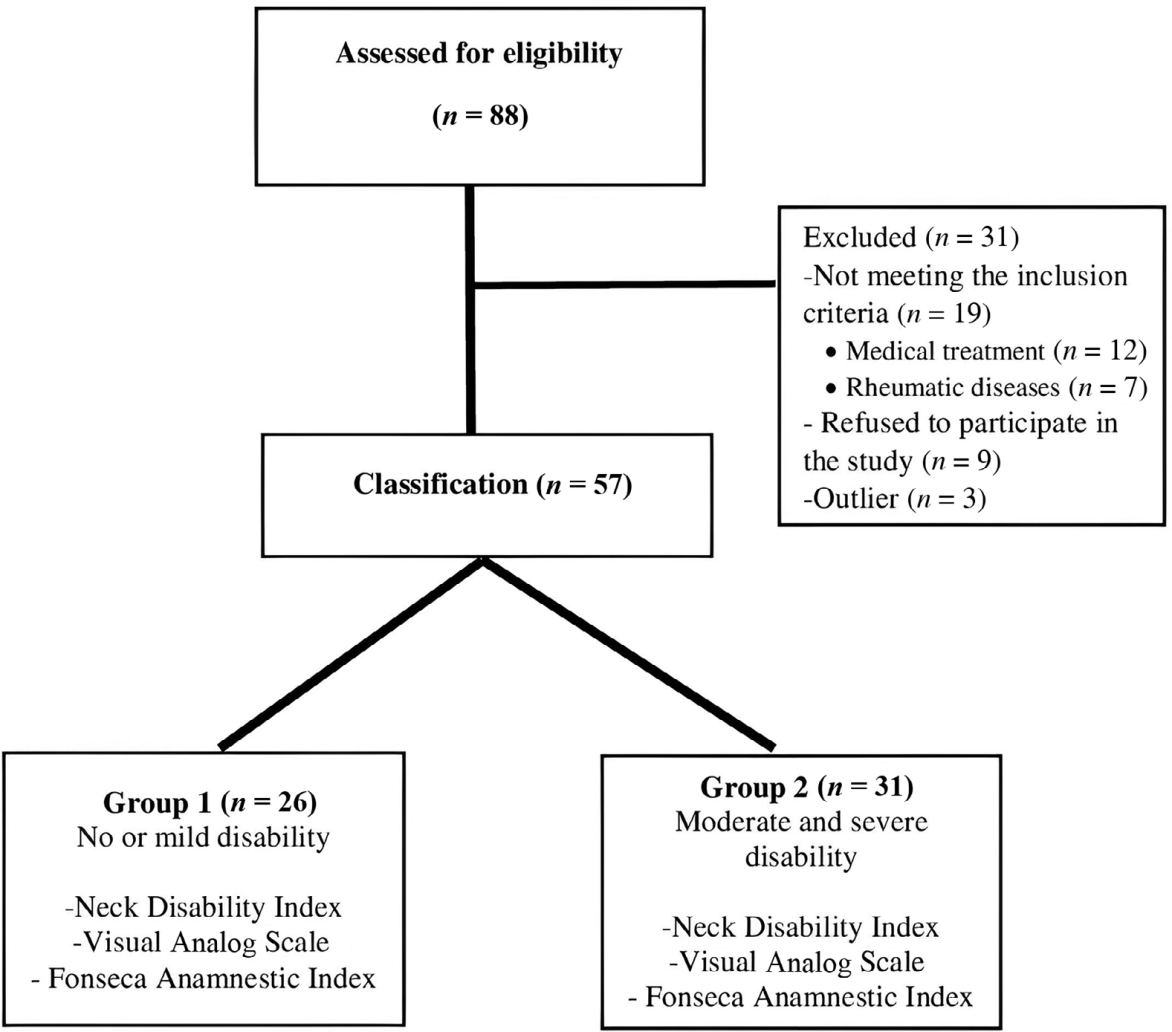
The participant selection process.

From May 2022 to September 2022, 88 patients were screened for eligibility, with 31 individuals excluded (Figure 1). Eligible participants were classified into two groups based on their Neck Disability Index (NDI) scores [20, 21]: No or mild neck disability (NDI score 0–14), or moderate‐to‐severe neck disability (NDI score ≥ 15).

### Outcomes

The primary clinical outcome measures were pain intensity, neck disability, and TMD severity, assessed as described in the following:

#### TMJ and neck pain intensity

The TMJ and the neck pain intensity in daily life were both measured using the Visual Analog Scale (VAS). Participants were asked about the average cervical and TMJ pain intensity experienced during the last month. Pain intensity was assessed by determining the point on a 10‐cm line where the person reported pain level. On the VAS, a score of 0 indicates no pain, whereas a score of 10 indicates unbearable pain [22]. Pain scores were individually recorded for both cervical and temporomandibular regions.

#### Neck Disability Index

The questionnaire consists of 10 items, including questions relating to pain intensity, personal care, lifting, reading, headaches, concentration, working, driving, sleeping, and leisure activities. Each item is scored on a six‐point scale, with 0 indicating no disability and 5 indicating full disability. The total score indicates the level of disability of the neck: 0–4 points means no disability, 5–14 points means mild disability, 15–24 points means moderate disability, 25–34 points means severe disability, and 35 points or above means total disability. Participants were divided into two groups based on their neck disability scores. The first group included individuals with no or mild neck disability (0–14 points), while the second group consisted of those with moderate‐to‐severe neck disability (above 15 points) [20, 21]. Telci et al. established the validity and reliability of the Turkish version of the NDI inventory [23].

#### Fonseca Anamnestic Index

The Fonseca Anamnestic Index (FAI) is reported as a reliable and valid index that determines the presence and severity of TMD. The reliability and validity of the Turkish version of FAI have been demonstrated to be excellent [24]. It consists of 10 items, each with 3 answer options: yes (10 points), sometimes (5 points), and no (0 points). A score of 0–15 indicates the absence of signs and symptoms of TMD, a score of 20–45 indicates mild TMD, a score of 50–65 indicates moderate TMD, and a score of 70–100 indicates severe TMD.

### Statistical analysis

The study used G*Power (v3.1) software to calculate sample size [25]. We assumed a two‑sided independent‑samples *t*‑test to detect a two‑point difference in TMJ pain intensity (VAS) between the no/mild and moderate/severe neck‑disability groups [26], assuming a common standard deviation (SD) of 2, α = 0.05, power = 0.95, and a 1:1 allocation. This resulted in a total sample size of 49; however, allowing for 10% attrition, the target sample size was set to 54 [27]. Statistical analyses were performed using r software (version 4.4.3; R Foundation for Statistical Computing).

Continuous variables (FAI, NDI total score, and pain intensity scores) are reported as mean values ± SD, and categorical variables (NDI category, sex, education level, income level, and marital status) are reported as frequencies (%). Between‐group comparisons of continuous outcomes (groups defined by NDI category: no/mild vs. moderate/severe) were performed using independent‐samples *t*‐tests; Welch's correction was applied when variances were unequal. The results are presented as group means, SDs, mean differences, and 95% confidence intervals (CIs).

We used multiple linear regression to examine the associations between the NDI total score and FAI score, respectively the TMJ pain score. The covariates considered were gender, age, education, income, marital status, and neck pain. All covariates were initially entered, then removed sequentially according to a prespecified parsimony rule: a covariate was removed if excluding it both altered the adjusted *R* ^2^ by less than 10%, and if it changed the NDI coefficient by less than 10%. The model fit is summarized using the adjusted *R* ^2^. Results are presented as regression coefficient values and their 95% CIs. The assumptions of linearity, normality, homoscedasticity, and multicollinearity were assessed using residual plots and variance inflation factors.

## RESULTS

### Participants

Participants were classified according to their neck disability scores into those with no or mild disability (*n* = 26, 69% women) and those with moderate to severe disability (*n* = 31, 85% women) (Table 1).

**TABLE 1:** Sociodemographic characteristics of the participants.

	Total, *n* = 57	No/mild neck disability, *n* = 26	Moderate/severe neck disability, *n* = 31
Age (years)			
18–35	14	7	7
36–55	35	15	20
56–65	8	4	4
Gender			
Female	45 (79%)	18 (69.2%)	27 (87.1%)
Male	12 (21%)	8 (30.8%)	4 (12.9%)
Education level (highest completed level)			
Primary school	18 (31.6%)	10 (38.5%)	8 (25.8%)
Secondary school	10 (17.5%)	4 (15.4%)	6 (19.4%)
High school	14 (24.6%)	4 (15.4%)	10 (32.2%)
Undergraduate	15 (26.3%)	8 (30.7%)	7 (22.6%)
Income level			
<Minimum wage^a^	16 (28%)	5 (19.2%)	11 (35.5%)
Minimum wage	24 (4.3%)	11 (42.3%)	13 (41.9%)
Double minimum wage	12 (21%)	6 (23.1%)	6 (19.4%)
>Three times the minimum wage	5 (8.8%)	4 (15.4%)	1 (3.2%)
Marital status			
Single	10 (17.5%)	3 (11.5%)	7 (22.6%)
Married	47 (82.5%)	23 (88.5%)	24 (77.4%)
	**Mean ± SD**		
Neck disability index score (mean ± SD)	15.58 ± 6.56	9.85 ± 2.60	19.97 ± 5.12

^a^ Minimum Wage: $455.

### TMD severity

TMD symptoms were present in 94.5% of the total sample. Participants with moderate/severe neck disability showed a higher prevalence and severity of TMD compared to participants with no/mild neck disability (100% vs. 88.5%). The mean difference in the FAI score between the moderate/severe neck disability group (mean score = 50, SD = 20) and no/mild neck disability group (mean score = 40, SD = 19) was 10, 95% CI (−0.2, 20.1). Table 2 shows that the number of individuals with temporomandibular dysfunction was higher among those with moderate/severe neck disability.

**TABLE 2:** Distribution of the participants in the two Neck Disability Index (NDI) groups according to their mean scores and classification according to the Fonseca Anamnestic Index for temporomandibular disorders (TMD).

		Fonseca classification of TMD, *n* (%)			
NDI group	Mean Fonseca score ± SD	No	Mild	Moderate	Severe
No/mild (*n* = 26)	40.4 ± 19.2	3 (100%)	13 (50%)	6 (32%)	4 (44%)
Moderate/severe (*n* = 31)	50.3 ± 18.9	0 (0%)	13 (50%)	13 (58%)	5 (56%)
Total	45.8 ± 19.5	3 (100%)	26 (100%)	19 (100%)	9 (100)

### Pain intensity in TMJ and neck

TMJ pain scores were greater among participants with moderate/severe neck disability (mean score = 4.6, SD = 2.7) than among those with no/mild disability (mean score = 2.4, SD = 2.0); mean difference = 2.2, 95% CI  (0.9, 3.5). Neck pain was also higher in the moderate/severe‑disability group (mean score = 6.9, SD = 1.5) than in the no/mild‑disability group (mean = 5.5, SD = 1.7); mean difference = 1.4, 95% CI (0.6, 2.3).

Multiple linear regression analysis of the FAI score as a function of the predictors (NDI total score, gender, and income) showed that each 1‑point increase in the NDI score was associated with an estimated 0.81‑point increase in the Fonseca score (95% CI [−0.11, 1.74], *p* = 0.083). Female gender was positively associated with higher Fonseca Index scores (*β* = 11.99, 95% CI [−0.49, 24.48], p = 0.046). Including income (four categories) improved model fit overall; however, the apparent effect was driven by a single category, with sparse data in the highest category (*n* = 5) and no consistent clinical gradient. Given limited interpretability and the risk of overfitting, we did not retain income in the most parsimonious model and report results without this factor. Sequentially removing VAS neck pain, age, education level, marital status, and age produced only small (5%–9%) changes in the NDI estimate or adjusted *R* ^2^ and did not alter the direction of the association; thus, we retained NDI and gender and excluded others (Table 3).

**TABLE 3:** Multiple linear regression of the Fonseca Anamnestic Index score as a function of the predictors (NDI, VAS‐Neck, age, gender, education level, income, marital status). Also given is the most parsimonious model explaining the data.

		Full model (adj *R* ^2^ = 0.20)		Reduced model (adj *R* ^2 ^= 0.12)	
Predictor		B	95% CI for B	B	95% CI for B
Intercept		23.83	(−7.54, 55.20)	24.31	(9.25, 39.39)
NDI (total score)		1.07	(0.03, 2.10)	0.81	(−0.11, 1.74)
VAS‐Neck		0.63	(−2.67, 3.92)	–	–
Age	18–35 years	Ref.		–	–
	36–55 years	−12.14	(−25.69, 1.41)	–	–
	56–65 years	−8.59	(−29.01, 11.84)	–	–
Gender	Man	Ref.			
	Woman	16.98	(−3.37, 30.58)	11.99	(−0.49, 24.48)
Highest completed education	Primary	Ref.		–	–
	Secondary	−8.87	(−24.72, 6.98)	–	–
	High school	−10.17	(−24.39, 4.05)	–	–
	Undergraduate	−3.71	(22.01, 14.59)	–	–
Income level	<Minimum	Ref		–	–
	Minimum	2.44	(−11.33, 16.22)	–	–
	2× minimum	−0.03	(−16.07, 16.01)	–	–
	3× minimum	28.70	(−5.39, 52.01)	–	–
Marital status	Married	Ref.		–	–
	Single	−1.27	(−15.29, 12.74)	–	–

Abbreviations: CI, confidence interval; NDI, Neck Disability Index; VAS, Visual Analog Scale.

Multiple linear regression analysis of the TMJ pain scores as a function of the predictors (NDI total score and gender) showed that each 1‐point increase in NDI was associated with a 0.18‐point increase in TMJ pain (95% CI [0.06, 0.30], p = 0.003). Gender showed a positive association (*β* = 1.46, 95% CI [−0.15, 3.06], *p* = 0.074). Removal of age, education level, income, and marital status had negligible effects on the NDI estimate (<5% change) and model fit; removal of education led to a modest reduction in adjusted *R* ^2^ but did not change conclusions; removal of VAS neck pain caused a small decrease in adjusted *R* ^2^ (0.232–0.221) while leaving the NDI–TMJ association directionally consistent and statistically stronger (Table 4).

**TABLE 4:** Multiple linear regression of the temporomandibular joint pain score (VAS score) as a function of the predictors (NDI, VAS‐Neck, age, gender, education level, income, marital status). Also given is the most parsimonious model.

		Full model (adj *R* ^2^ = 0.30)		Reduced model (adj *R* ^2 ^= 0.22)	
Predictor		B	95% CI for B	B	95% CI for B
Intercept		−4.42	(−8.44, −0.40)	−0.29	(−2.23, 1.64)
NDI (total score)		0.15	(0.02, 0.29)	0.18	(0.06, 0.30)
VAS‐Neck		0.21	(−0.21, 0.63)	–	–
Age	18–35 years	Ref.		–	–
	36–55 years	1.47	(−0.26, 3.21)	–	–
	56–65 years	1.52	(−1.10, 4.13)	–	–
Gender	Man	Ref.			
	Woman	0.70	(−1.04, 2.44)	1.46	(−0.15, 3.06)
Highest completed education	Primary	Ref.		–	–
	Secondary	1.56	(−0.47, 3.59)	–	–
	High school	1.25	(−0.57, 3.07)	–	–
	Undergraduate	3.31	(0.97, 5.66)	–	–
Income level	<Minimum	Ref		–	–
	Minimum	−0.59	(−2.36, 1.17)	–	–
	2× minimum	−0.94	(−2.99, 1.12)	–	–
	3× minimum	−1.94	(−4.92, 1.05)	–	–
Marital status	Married	Ref.		–	–
	Single	2.45	(−0.66, 4.25)	–	–

Abbreviations: CI, confidence interval; NDI, Neck Disability Index; VAS, Visual Analog Scale.

## DISCUSSION

The findings reported here indicate that nearly all individuals diagnosed with cervical disc herniation also presented with TMDs, and gender and higher NDI scores were associated with greater TMD severity. This finding aligns with previous research reporting a TMD prevalence of 46%–90% among individuals with neck pain, depending on the assessment methods used [4, 28]. The coexistence of TMD and cervical disc herniation may be explained by shared muscular and fascial structures linking the cervical and mandibular regions, which can promote reciprocal dysfunction in musculoskeletal conditions [10, 29]. Forward‐head posture—common in cervical disc herniation—may further disrupt mandibular biomechanics and contribute to TMD symptoms through compensatory changes in the cervical and craniofacial musculature [30, 31, 32]. TMD, when left undiagnosed, can exacerbate cervical symptoms such as chronic neck pain, postural imbalance, and neuromuscular fatigue. It may also induce nonphysiological spinal adaptations and muscular dysfunction, worsening overall functional status [33, 34]. These findings underscore the importance of adopting a bidirectional clinical approach, where both cervical and temporomandibular regions are systematically assessed [18, 35]. Such an integrated perspective may enhance diagnostic accuracy and promote more effective, patient‐centered rehabilitation strategies, ultimately improving clinical outcomes and quality of life [36].

In addition to the high co‐occurrence of these disorders, our findings showed that TMJ pain intensity was significantly higher among individuals with greater neck disability. This finding was particularly evident among female participants, reinforcing the multifactorial and gender‐sensitive nature of TMD pain. It is noteworthy that the classification of participants according to neck disability levels, as measured by the NDI, resulted in more clinically informative associations with TMJ pain and TMD symptom severity than consideration of neck pain intensity alone. This aligns with prior research suggesting that biomechanical disability and central modulation, rather than cervical nociception, may be more relevant in chronic orofacial pain [12, 37, 38].

Mechanistically, increased neck disability may impair neuromuscular coordination by disrupting motor unit recruitment and the balance between synergistic and antagonistic muscle groups [39]. This may compromise motor control in the cervical and craniofacial systems, leading to maladaptive movement patterns and functional limitations [39, 40]. Additionally, chronic neck pain is often accompanied by central sensitization, such as trigeminal hyperalgesia and increased masticatory muscle sensitivity [41, 42], both of which may exacerbate TMD symptoms [43]. From a clinical standpoint, these interrelated mechanisms underscore the need for a dual‐region rehabilitation approach. Fragmented care that targets only the cervical or the masticatory system may fail to address the full scope of dysfunction. Incorporating routine TMD screening into cervical disc herniation management—especially in females and those with high NDI scores—could improve treatment outcomes. Implementing interdisciplinary, patient‐centered protocols that address both TMJ and cervical dysfunctions may lead to more effective and sustainable clinical outcomes.

This study has several limitations that should be considered when interpreting the findings. First, the sample size was not sufficient to allow for subgroup analysis based on the type or level of cervical disc herniation, and this may have limited the specificity of our conclusions. Second, the absence of a healthy control group restricts our ability to distinguish whether the observed TMD symptoms are unique to the cervical disc herniation population or reflect a more generalized pattern. Although we attempted to control for potential confounders such as gender and age, the cross‐sectional design inherently limits causal interpretations and may be subject to residual confounding. Lastly, while the use of standardized questionnaires ensures consistency and feasibility in large samples, it may not capture the full clinical complexity of individual cases. Nevertheless, despite these limitations, the consistency of associations observed, and the statistical robustness of the models support the relevance of our findings. Future research should aim to address these limitations through larger, longitudinal designs incorporating clinical assessments and control groups.

These findings presented here support the integration of routine TMD assessment into the evaluation of cervical disc herniation patients, particularly those with moderate‐to‐severe neck disability. Addressing TMD in this population may prevent chronic symptom cycles and enhance rehabilitation outcomes. Future studies should explore causal relationships through longitudinal designs and assess the efficacy of integrated treatment protocols.

## AUTHOR CONTRIBUTIONS

**Conceptualization**: Turgay Altunalan, Esin Geçgil Nazli, and Ilayda Gür. **Investigation**: Esin Geçgil Nazli and Ilayda Gür. **Methodology**: Turgay Altunalan**. Data curation**: Turgay Altunalan, Esin Geçgil Nazli, and Ilayda Gür. **Formal analysis**: Turgay Altunalan and Nurhayat Korkmaz Ücünçü. **Writing—original draft**: Esin Geçgil Nazli, Ilayda Gür, and Nurhayat Korkmaz Ücünçü. **Writing—review and editing**: Turgay Altunalan and Nurhayat Korkmaz Ücünçü.

## CONFLICT OF INTEREST STATEMENT

The authors declare no conflicts of interest.

## References

[1] Sharrak S , Al Khalili Y . Cervical disc herniation. In: StatPearls. Treasure Island, FL: StatPearls Publishing; 2023.

[2] Wong JJ , Côté P , Quesnele JJ , Stern PJ , Mior SA . The course and prognostic factors of symptomatic cervical disc herniation with radiculopathy: a systematic review of the literature. Spine J. 2014;14:1781‐9. 24614255 10.1016/j.spinee.2014.02.032

[3] Çebi AT . Temporomandibular Eklem Disfonksiyonlu Hastalarda Servikal Disk Hernisi Görülme Sıklığının Degerlendirilmesi. Turkiye Klinikleri. Dishekimligi Bilimleri Dergisi. 2020;26:318‐22.

[4] Subaşı SS , Gelecek N , İlçin N , Çeliker Ö Servikal Disk Hernili Hastalarda Temporomandibular Eklem Disfonksiyonu Görülme Sıklığı. Türk Plastik Rekonstrüktif ve Estetik Cerrahi Dergisi. 2012;19:125‐30.

[5] Wilkie G , Al‐Ani Z . Temporomandibular joint anatomy, function and clinical relevance. Br Dent J. 2022;233:539‐46. 36241801 10.1038/s41415-022-5082-0

[6] Valesan LF , Da‐Cas CD , Réus JC , Denardin ACS , Garanhani RR , Bonotto D , et al. Prevalence of temporomandibular joint disorders: a systematic review and meta‐analysis. Clin Oral Investig. 2021;25:441‐53. 10.1007/s00784-020-03710-w 33409693

[7] Kapos FP , Exposto FG , Oyarzo JF , Durham J . Temporomandibular disorders: a review of current concepts in aetiology, diagnosis and management. Oral Surg. 2020;13:321‐34. 34853604 10.1111/ors.12473 PMC8631581

[8] Güzel HÇ , Aracı A , Telci EA , Cımbız A . Evaluation of temporomandibular joint dysfunction in patients with chronic neck pain. Int J Tradition Complement Med Res. 2022;3:117‐24.

[9] Matheson EM , Fermo JD , Blackwelder RS . Temporomandibular Disorders: Rapid Evidence Review. Am Fam Physician. 2023;107:52‐58. 36689971

[10] Walczyńska‐Dragon K , Baron S , Nitecka‐Buchta A , Tkacz E . Correlation between TMD and cervical spine pain and mobility: is the whole body balance TMJ related? BioMed Res Int. 2014;2014:582414. 10.1155/2014/582414 25050363 PMC4090505

[11] Rocha C , Croci C , Caria P . Is there relationship between temporomandibular disorders and head and cervical posture? A systematic review. J Oral Rehabil. 2013;40:875‐81. 24118029 10.1111/joor.12104

[12] de Oliveira‐Souza AIS , JKdO F , Barros MM , de Oliveira DA . Cervical musculoskeletal disorders in patients with temporomandibular dysfunction: a systematic review and meta‐analysis. Bodyw Mov Ther. 2020;24:84‐101. 10.1016/j.jbmt.2020.05.001 33218570

[13] Silveira A , Gadotti IC , Armijo‐Olivo S , Biasotto‐Gonzalez D , Magee D . Jaw dysfunction is associated with neck disability and muscle tenderness in subjects with and without chronic temporomandibular disorders. BioMed Res Int. 2015;2015:512792. 10.1155/2015/512792 25883963 PMC4391655

[14] Fonseca JB , Lima VCN , Santiago JA , Lima FAP . Occurrence and severity of neck disability in individuals with different types of temporomandibular disorder. J Oral Maxillofac. 2021;25:471‐6. 10.1007/s10006-021-00943-1 33527258

[15] Szarejko KD , Gołębiewska M , Lukomska‐Szymanska M , Kuć J . Stress experience, depression and neck disability in patients with temporomandibular disorder—myofascial pain with referral. J Clin Med. 2023;12:1988. 10.3390/jcm12051988 36902775 PMC10004681

[16] Tavares LF , Gadotti IC , Carvalho BG , Fernandes APM , Padilha Silva J , Barbosa GAS , et al. Are neck pain, disability, and deep neck flexor performance the same for the different types of temporomandibular disorders? Cranio. 2024;42:770‐8. 10.1080/08869634.2022.2052582 35300577

[17] Thorp JN , Willson J . The Neck Disability Index is not correlated with some parameters of temporomandibular disorders: a cross‐sectional study. J Oral Facial Pain Headache. 2019;33:39–46. 30129940 10.11607/ofph.1992

[18] Ghodrati M , Mosallanezhad Z , Shati M , Noroozi M , Moghadam AN , Rostami M , et al. Adding temporomandibular joint treatments to routine physiotherapy for patients with non‐specific chronic neck pain: a randomized clinical study. Bodyw Mov Ther. 2020;24:202‐12. 10.1016/j.jbmt.2019.11.004 32507146

[19] Von Elm E , Altman DG , Egger M , Pocock SJ , Gøtzsche PC , Vandenbroucke JP . The Strengthening the Reporting of Observational Studies in Epidemiology (STROBE) statement: guidelines for reporting observational studies. Lancet. 2007;370:1453‐7. 18064739 10.1016/S0140-6736(07)61602-X

[20] Xie Y , Thomas L , Johnston V , Coombes BK . Cervical and axioscapular muscle stiffness measured with shear wave elastography: a comparison between different levels of work‐related neck disability. J Electromyogr Kinesiol. 2023;69:102754. 10.1016/j.jelekin.2023.102754 36773478

[21] MacDermid JC , Walton DM , Avery S , Blanchard A , Etruw E , Mcalpine C , et al. Measurement properties of the neck disability index: a systematic review. J Orthop Sports Phys Ther. 2009;39:400‐17. 19521015 10.2519/jospt.2009.2930

[22] Tuncer A , Ergun N , Karahan S . Temporomandibular disorders treatment: comparison of home exercise and manual therapy. Fizyoter Rehabil. 2013;24: 9–16.

[23] Telci EA , Karaduman A , Yakut Y , Aras B , Simsek IE , Yagli N . The cultural adaptation, reliability, and validity of neck disability index in patients with neck pain: a Turkish version study. Spine. 2009;34:1732‐5. 19770615 10.1097/BRS.0b013e3181ac9055

[24] Kaynak BA , Taş S , Salkın Y . The accuracy and reliability of the Turkish version of the Fonseca Anamnestic Index in temporomandibular disorders. Cranio. 2023;41:78‐83. 32840464 10.1080/08869634.2020.1812808

[25] Faul F , Erdfelder E , Lang A‐G , Buchner A . G* Power 3: a flexible statistical power analysis program for the social, behavioral, and biomedical sciences. Behav Res Methods. 2007;39:175‐91. 17695343 10.3758/bf03193146

[26] Son J , Kim ES , Lee YJ , Lee NW , Ha IH . Minimum clinically important difference and substantial clinical benefit in patients with chronic temporomandibular disorders. J Oral Rehabil. 2024;51:1468‐74. 38706163 10.1111/joor.13717

[27] Calixtre LB , Oliveira AB , Alburquerque‐Sendín F , Armijo‐Olivo S . What is the minimal important difference of pain intensity, mandibular function, and headache impact in patients with temporomandibular disorders? Clinical significance analysis of a randomized controlled trial. Musculoskelet Sci Pract. 2020;46:102108. 10.1016/j.msksp.2020.102108 31999615

[28] Sohail M , Ashraf HS , Shahzad Z , Anjum A . Frequency of temporomandibular dysfunction, pain intensity and joint s level of disability among patients with cervical pain under physical therapy treatment in Lahore, Pakistan. Rawal Med J. 2021;46:114‐17.

[29] Visscher CM , Lobbezoo F , De Boer W , Van Der Zaag J , Naeije M . Prevalence of cervical spinal pain in craniomandibular pain patients. Eur J Oral Sci. 2001;109:76‐80. 11347659 10.1034/j.1600-0722.2001.00996.x

[30] Grondin F , Hall T , Laurentjoye M , Ella B . Upper cervical range of motion is impaired in patients with temporomandibular disorders. Cranio. 2015;33:91‐9. 25919749 10.1179/0886963414Z.00000000053

[31] Ohmure H , Miyawaki S , Nagata J , Ikeda K , Yamasaki K , Al‐Kalaly A . Influence of forward head posture on condylar position. J Oral Rehabil. 2008;35:795‐800. 18808377 10.1111/j.1365-2842.2007.01834.x

[32] Siu WS , Shih YF , Lee SY , Hsu CY , Wei MJ , Wang TJ , et al. Alterations in kinematics of temporomandibular joint associated with chronic neck pain. J Oral Rehabil. 2022;49:860‐71. 35699317 10.1111/joor.13347

[33] Lu G , Du R . Temporomandibular joint disorder: an integrated study of the pathophysiology, neural mechanisms, and therapeutic strategies. Arch Oral Biol. 2024;164:106001. 10.1016/j.archoralbio.2024.106001 38749387

[34] Raciti L , Ferrillo M , Ammendolia A , Raciti G , Curci C , Calafiore D , et al. Neurophysiological examination for the diagnosis of orofacial pain and temporomandibular disorders: a literature review. Diagnostics. 2025;15:1035. 10.3390/diagnostics15081035 40310423 PMC12026286

[35] Rezaie K , Amiri A , Takamjani EE , Shirani G , Salehi S , Alizadeh L . The efficacy of neck and temporomandibular joint (TMJ) manual therapy in comparison with a multimodal approach in the patients with TMJ dysfunction: a blinded randomized controlled trial. Med J Islam Repub Iran. 2022;36:45. 10.47176/mjiri.36.45 36128309 PMC9448471

[36] de Silva ALC , da Silva BOM , Cabral LN . Prevalence of cervical alterations in patients with temporomandibular dysfunction in a specialized referral service. Res Soc Dev. 2022;11:e560111033294. 10.33448/rsd-v11i10.33294

[37] dos Santos GJR , Mendes LMR , Menezes LDO , Grossi DB , Folha GA . Relationships between craniofacial pain and disability, neck disability, and orofacial myofunctional condition in patients with temporomandibular dysfunction. Headache Med. 2024;15:44. https://headachemedicine.com.br/index.php/hm/article/view/1149

[38] Mendes LMR , Marçal JCS , Menezes LDO , Grossi DB . Craniofacial and Neck disability predict the presence of symptoms related to central sensitization in individuals with temporomandibular disorders. Headache Med. 2024;15:32. https://headachemedicine.com.br/index.php/hm/article/view/1123

[39] Farina D , Arendt‐Nielsen L , Merletti R , Graven‐Nielsen T . Effect of experimental muscle pain on motor unit firing rate and conduction velocity. J Neurophysiol. 2004;91:1250‐9. 14614105 10.1152/jn.00620.2003

[40] Armıjıo‐Olıvo S , Magee D . Electromyographic activity of the masticatory and cervical muscles during resisted jaw opening movement. J Oral Rehabil. 2007;34:184‐94. 17302946 10.1111/j.1365-2842.2006.01664.x

[41] Catanzariti J‐F , Debuse T , Duquesnoy B . Chronic neck pain and masticatory dysfunction. Joint Bone Spine. 2005;72:515‐9. 16226475 10.1016/j.jbspin.2004.10.007

[42] La Touche R , Fernández‐de‐Las‐Peñas C , Fernández‐Carnero J , Díaz‐Parreño S , Paris‐Alemany A , Arendt‐Nielsen L . Bilateral mechanical‐pain sensitivity over the trigeminal region in patients with chronic mechanical neck pain. J Pain. 2010;11:256‐63. 19945351 10.1016/j.jpain.2009.07.003

[43] Quartana PJ , Finan PH , Smith MT . Evidence for sustained mechanical pain sensitization in women with chronic temporomandibular disorder versus healthy female participants. J Pain. 2015;16:1127‐35. 26281948 10.1016/j.jpain.2015.08.002

